# NMR refinement and peptide folding using the GROMACS software

**DOI:** 10.1007/s10858-021-00363-z

**Published:** 2021-03-28

**Authors:** Anna Sinelnikova, David van der Spoel

**Affiliations:** 1 Department of Physics and Astronomy, Uppsala University, Uppsala, Sweden; 2 Department of Cell and Molecular Biology, Uppsala University, Uppsala, Sweden

**Keywords:** Python, NMR-STAR, Force Field, Amber, Charmm

## Abstract

Nuclear magnetic resonance spectroscopy is used routinely for studying the three-dimensional structures and dynamics of proteins and nucleic acids. Structure determination is usually done by adding restraints based upon NMR data to a classical energy function and performing restrained molecular simulations. Here we report on the implementation of a script to extract NMR restraints from a NMR-STAR file and export it to the GROMACS software. With this package it is possible to model distance restraints, dihedral restraints and orientation restraints. The output from the script is validated by performing simulations with and without restraints, including the *ab initio* refinement of one peptide.

## Introduction

Nuclear Magnetic Resonance spectroscopy is a powerful technique to study structure and dynamics of biologically relevant molecules in solution (Palmer 2004; Kay 2016). Due to steady methodological progress, membrane proteins (Opella and Marassi 2017) as well as disordered proteins (Gibbs et al. 2017) and even macromolecules *in vivo* (Inomata et al. 2009; Sakakibara et al. 2009; Luchinat and Banci 2017) can now be studied using NMR spectroscopy techniques. Molecular dynamics simulations have been used for over thirty years as a tool to supplement the sometimes limited amounts of data, and to allow determination and refinement of structures or aid the interpretation of experimental data (Torda and Van Gunsteren 1991; Torda et al. 1993). In addition, NMR data can be used to validate simulation results giving detailed insights when simulated structures deviate from experimental data (van der Spoel and Lindahl 2003; Lange et al. 2010) or to validate force fields (Hornak et al. 2006; Huang and MacKerell 2013). Determination of biomolecular structures is to a large extent automated these days (Wrz et al. 2017). Nevertheless, it may be advantageous to both the NMR and the simulations communities to have a variety of tools to analyze biomolecules using NMR data. Therefore we have implemented a script to include restraints from NMR into the GROMACS software suite for classical molecular dynamics simulations(Berendsen et al. 1995; Lindahl et al. 2001; van der Spoel et al. 2005; Hess et al. 2008; Pronk et al. 2013; Páll et al. 2015). The script is validated by performing restrained as well as unrestrained molecular dynamics simulations of peptides from the Protein Data Bank (Westbrook et al. 2002) and by performing *ab initio* refinement of a short peptide.

## Theory

### Background

Here, we briefly recap relevant equations that are implemented in the GROMACS software suite. Within classical molecular simulation software packages, trajectories of molecules can be simulated by numerically solving of Newton’s equations of motion (Allen and Tildesley 1987). To do so, the force at every atom is calculated as the negative gradient of the potential function. The potential functions in turn, are divided into three different categories:

- bonded forces, including chemical bonds, angles and dihedrals,
- Van der Waals and Coulomb forces,
- different kind of restraints.

In this paper we are interested in the last group, using the restraint information that can be obtained from NMR experiments. We consider the three types of restraints that are implemented in GROMACS: distance, dihedral and orientation restraints.

Distance restraints introduce a lower and upper limit for the distance for a particular atom pair. In GROMACS this is implemented as a flat-bottom harmonic oscillator potential:

1 $$\begin{aligned} V_{dr}(r_{ij}) = {\left\{ \begin{array}{ll} \frac{1}{2}k_{dr}(r_{ij}-r_0)^2, &{} \text{ for } r_{ij}<r_0\\ 0, &{} \text{ for } r_0\le r_{ij}<r_1\\ \frac{1}{2}k_{dr}(r_{ij}-r_1)^2, &{} \text{ for } r_1 \le r_{ij}<r_2\\ \frac{1}{2}k_{dr}(r_2-r_1)(2r_{ij} - r_2 - r_1), &{} \text{ for } r_2 \le r_{ij} \end{array}\right. } \end{aligned}$$

where $r_{ij}$ is a distance between atoms *i* and *j*, $k_{dr}$ is a distance restraint force constant, $r_0$ is a lower bound of the restraint and $r_{1}$ and $r_{2}$ are two upper bounds. The second upper bound is introduced to prevent extremely large forces in case an atom pair is far from the target distance. In addition, GROMACS implements time averaging (Torda et al. 1989) as well as ensemble averaging of distance restraints.

Dihedral angles can be restrained using a similar flat-bottom potential:

2 $$\begin{aligned} V_{dihr}(\phi ') = {\left\{ \begin{array}{ll} \frac{1}{2}k_{dihr}(\phi ' - \phi _0 - \varDelta \phi )^2, &{} \text{ for } \phi ' > \varDelta \phi \\ 0, &{} \text{ for } \phi ' \le \varDelta \phi \end{array}\right. } \end{aligned}$$

where

3 $$\begin{aligned} \phi ' = (\phi - \phi _0)\,\mathrm{MOD}\, 2\pi \end{aligned}$$

with $\phi _0$ the reference angle, typically derived from J-coupling constants using a Karplus relation (Karplus 1959). Time averaging can be applied for dihedral restraints (Torda et al. 1993) in GROMACS as well (Lindahl et al. 2020).

Orientation restraints can be obtained from e.g. residual dipolar couplings. They have been implemented in GROMACS previously, including time and ensemble averaging (Hess and Scheek 2003). We refer to that paper or the GROMACS manual (Lindahl et al. 2020) for more information because the mathematics is rather extensive.

### Implementation details

Here we briefly describe the script “nmr2gmx.py” used to convert a NMR-STAR file (Ulrich et al. 2019) to GROMACS inputs. The NMR-STAR file format is supported by a number of software packages and is the standard for storing processed NMR data. For the purpose of this project it is important to note that there is a Python library that can be used to read and process the content of the files (Wedell and Baskaran 2020).

The scripts use two layers of conversion of inputs. First, from NMR notation for degenerate groups to actual atoms, and second, to convert to atom names matching the force fields. The latter is needed since, unfortunately, at least three different naming schemes for hydrogen atoms are in use today despite that standard nomenclature (IUPAC-IUB 1970) predates biomolecular force fields. Table  1 lists the effective translations. The script expands, for instance, the interaction between an Ala MB and an Ile MD to 9 distances that are however treated as one restraint using $r^{-6}$ averaging of the distances. Logical OR statements in the input for distance restraints are honored. Both dihedral restraints and orientation restraints apply the renaming conventions in Table 1. In the case of dihedral restraints, the lower and upper bounds are extracted from the data and the average angle is computed, taking periodicity into account. A harmonic potential is applied starting from the bounds (Eqn. 2). For orientation restraints the chemical shift anisotropy $\delta$ is read from the NMR data and output to GROMACS format (Lindahl et al. 2020). Multiple chains are supported as well. More documentation for the script is at the GitHub repository (Sinelnikova et al. 2020).

**Table 1:** Atom and identifier name translations applied in the script for amino acids and nucleobases

Residue	Identifier	Amber	Charmm
Backbone	H	H	HN
Ala	MB	HB$_{1,2,3}$	HB$_{1,2,3}$
Thr	MG	HG$_{1,2,3}$	HG$_{1,2,3}$
Ile	MG	HG2$_{1,2,3}$	HG2$_{1,2,3}$
Ile	HG1$_2$	HG1$_1$	HG1$_1$
Ile	HG1$_3$	HG1$_2$	HG1$_2$
Ile	MD	HD$_{1,2,3}$	HD$_{1,2,3}$
Ile	CD1	CD	CD
Ile	HD1$_x$	HD$_x$	HD$_x$
Gly	HA2	HA1	HA1
Gly	HA3	HA2	HA2
Ser, Thr, Cys	HG	HG	HG$_1$
Lys, Asn, Ser, Asp, Glu, Pro, Gln, Arg, Met, Trp, Tyr, Phe, His, Leu	HB$_2$	HB$_1$	HB$_1$
Lys, Asn, Ser, Asp, Glu, Pro, Gln, Arg, Met, Trp, Tyr, Phe, His, Leu	HB$_3$	HB$_2$	HB$_2$
Lys, Asn, Ser, Asp, Glu, Pro, Gln, Arg, Met, Val	HG$_3$	HG$_2$	HG$_2$
Lys, Asn, Ser, Asp, Glu, Pro, Gln, Arg, Met, Val	HG$_3$	HG$_2$	HG$_2$
Lys, Asn, Ser, Asp, Glu, Pro, Gln, Arg, Met	HD$_3$	HD$_2$	HD$_2$
Lys, Asn, Ser, Asp, Glu, Pro, Gln, Arg, Met	HD$_3$	HD$_2$	HD$_2$
Lys, Asn, Ser, Asp, Glu, Pro, Gln, Arg, Met	HE$_3$	HE$_2$	HE$_2$
Lys, Asn, Ser, Asp, Glu, Pro, Gln, Arg, Met	HE$_3$	HE$_2$	HE$_2$
Met	ME	HE$_{1,2,3}$	HE$_{1,2,3}$
Lys	QZ	HZ$_{1,2,3}$	HZ$_{1,2,3}$
Arg	QH1	HH1$_{1,2}$	HH1$_{1,2}$
Arg	QH2	HH2$_{1,2}$	HH2$_{1,2}$
Leu	MD1	HD1$_{1,2,3}$	HD1$_{1,2,3}$
Leu	MD2	HD2$_{1,2,3}$	HD2$_{1,2,3}$
Tyr, Phe	QD	HD$_{1,2}$	HD$_{1,2}$
Tyr, Phe	QE	HE$_{1,2}$	HE$_{1,2}$
G, A, U, C, DG, DA, DT, DC	HO2’	HO’2	HO’2
G, A, U, C, DG, DA, DT, DC	H5’	H5’1	H5’1
G, A, U, C, DG, DA, DT, DC	H5”	H5’2	H5’2
G, A, U, C, DG, DA, DT, DC	H2’	H2’1	H2’1
G, A, U, C, DG, DA, DT, DC	H2”	H2’2	H2’2
DT	M7	H7$_{1,2,3}$	H7$_{1,2,3}$

## Methods

### Technical validation

A test set is part of the package. In short, 44 PDB entries are downloaded and processed and the resulting output files compared to reference tables (that is, the GROMACS input files). If input files are indeed correct, a GROMACS energy minimization is run and the output structure compared to the PDB structure. Since the energy minimization is performed *in vacuo* some conformational changes does occur but in all cases the root mean square deviation remains within 0.02 nm. By applying the scripts to a few dozen different entries, it was possible to detect potential errors. If the script is updated or extended in the future the test set can be used to make sure functionality remains intact. The test set includes systems containing proteins, RNA and DNA and those biomolecules supported by the Amber force field should work with the script as well. In systems where GROMACS does not recognize e.g. the protonation state of Histidine residues, a warning is printed and one or more restraints may be skipped.

### Simulation details

Several short polypeptides were taken from the Protein Data Bank to make a full MD run with and without restraints and thus verify the compatibility of the output from the program with the GROMACS software. The peptides are of different lengths and have different types of restraints. Table 2 lists the polypeptides used, their lengths in number of residues and what type of restraints were obtained from NMR data file for each. All proteins were simulated for 20 ns in a cubic water box with periodic boundary conditions at temperature equals 300 K. Particle mesh-Ewald summation (Darden et al. 1993; Essmann et al. 1995) was used to treat long-range Coulomb interactions, while Lennard-Jones interactions were cut-off at 1 nm with analytical tail corrections for the long range dispersion (Allen and Tildesley 1987). Whether or not such approximations will be acceptable in the future is under scrutiny right now (van der Spoel et al. 2020). Temperature was controlled using the Bussi thermostat (Bussi et al. 2007) with a time constant of 0.5 ps, while pressure was maintained at 1 bar using the Parrinello-Rahman algorithm (Parrinello and Rahman 1981) with a time constant of 2 ps. The Amber99SB-ILDN (Cornell et al. 1995; Lindorff-Larsen et al. 2011) force field was used in combination with the tip3p water model (Jorgensen et al. 1983) to perform MD simulations.

The Charmm force field version 27 (MacKerell et al. 1998; Foloppe and MacKerell 2000) as implemented in GROMACS is supported as well in v1.0 of the program although the support in GROMACS is somewhat more limited than for Amber and therefore there are only 28 test cases. Other force fields can readily be implemented in the script and guidelines for this can be found in README file on the GitHub page(Sinelnikova et al. 2020).

### Analysis

Apart from inspecting the restraints, we compute the root mean square deviation of distances (RMSD) from the simulation trajectories as follows. All the atom-atom distances $r_{ij}^{MD}$ in a protein are computed at each time in the simulation and the distances are compared to the corresponding $r_{ij}^{NMR}$ in the experimental references structure. The RMSD is then computed as the root mean square difference between $r_{ij}^{MD}$ and $r_{ij}^{NMR}$. The advantage of this method over positional RMSD is that the superposition step is omitted, which may lead to arbitrary jumps in RMSD due to small changes in coordinates if the protein structures differ a lot. Since multiple experimental models are available for all the proteins (Table 2), we compute the RMSD to each of the models at each time point in the simulation and then take the lowest value. The rationale behind this is that the experimental structures are equally likely, and if the simulated protein is close to any of the structures, the deviation is low.

**Table 2:** Proteins used for the MD simulations with number of residues and number of distance (#disres), dihedral (#dihres) respectively orientation (#orires) restraints obtained from corresponding NMR data files

PDB ID	#residues	#disres	#dihres	#orires	#models
6cj8 (Yang et al. 2018)	17	577			20
2luf (Bathula et al. 2013)	20	398			10
2leu (Fregeau Gallagher et al. 1997)	37	420			18
1lb0 (Biron et al. 2002)	13	202	9		1
2md6 (Lebbe et al. 2014)	18	59	10		15
1qqv (Vardar et al. 1999)	67	327	19		1
1d3z (Cornilescu et al. 1998)	78	2727	98	62	10
1lvz (Koenig et al. 2002)	11	121	12	8	20

Number of experimental models

## Results and discussion

### Evaluation of distance restraint parameters

Restrained simulations require a number of parameters like the force constant $k_{dr}$ (Eqn. 1) and the constant $\tau _{dr}$ used for time averaging (Torda et al. 1989). A number of different combinations of these parameters were evaluated to find values that work well in most cases. Table 3 lists the distance violations averaged over 20 ns simulations of first 6 proteins in Table 2. Based on this result we recommend a force constant $k_{dr}$ of 1000 kJ mol$^{-1}$ nm$^{-2}$ and an averaging time $\tau _{dr}$ of 500 ps. It should be noted that the optimal values for these parameters depend on a number of factors, such as peptide length and flexibility and indeed how well folded the peptide is to start with.

**Table 3:** Evaluation of the effect of force constant $k_{dr}$ (kJ mol$^{-1}$ nm$^{-2}$) and averaging time $\tau _{dr}$ (ps) on average distance violations (nm) averaged over 6 proteins

	$$k_{dr}$$			
$\tau _{dr}$	10	100	500	1000
0	$0.020 \pm 0.010$	$0.015 \pm 0.009$	$0.010 \pm 0.007$	$0.008 \pm 0.006$
10	$0.026 \pm 0.009$	$0.014 \pm 0.009$	$0.010 \pm 0.006$	$0.008 \pm 0.005$
100	$0.021 \pm 0.010$	$0.015 \pm 0.009$	$0.008 \pm 0.005$	$0.005 \pm 0.002$
500	$0.020 \pm 0.010$	$0.014 \pm 0.008$	$0.006 \pm 0.003$	$0.004 \pm 0.002$
1000	$0.022 \pm 0.010$	$0.014 \pm 0.008$	$0.007 \pm 0.003$	$0.004 \pm 0.002$

### Validation

Table 4 presents a comparison of the distance, dihedral and orientation violations together with distance RMSD (see section 3.3), in simulations with and without restraints using the recommended set of distance restraints parameters according Table 3: $\kappa _{dr} = 1000$ kJ mol$^{-1}$ nm$^{-2}$ and $\tau _{dr} = 500$ ps. For all types of restraints the average violations are quite a bit lower with restraints turned on, showing that the potentials are effective. The same tendency can be seen for the distance RMSD in some simulations (2leu, 1lb0, 1lvz): without restraints the deviations are higher than with restraints. For the other proteins the difference in RMSD is within the uncertainty.

**Table 4:** Comparison table for violation of distance, dihedral and orientation restraints together with RMSD distance average for all simulated proteins

PDB ID	Distance (nm)		Dihedral ($^\circ$)		Orientation (Hz)		RMSDist (nm)	
	With	Without	With	Without	With	Without*	With	Without
6cj8	0.014	0.071	–	–	–	–	0.14 ± 0.05	0.14 ± 0.02
2luf	0.001	0.016	–	–	–	–	0.14 ± 0.05	0.17 ± 0.02
2leu	0.001	0.012	–	–	–	–	0.29 ± 0.08	0.71 ± 0.02
1lb0	0.002	0.002	0	5.03	–	–	0.17 ± 0.04	0.24 ± 0.05
2md6	0.002	0.012	0	0.20	–	–	0.11 ± 0.02	0.115 ± 0.009
1qqv	0.005	0.007	0	10.7	–	–	0.18 ± 0.02	0.20 ± 0.03
1d3z	0.001	0.002	6.12	15.0	0.41	0.94	0.085 ± 0.009	0.08 ± 0.02
1lvz	0.001	0.011	0.01	8.40	0.28	0.43	0.07 ± 0.02	0.15 ± 0.04

.“With” indicates that all restraints are taken into account, while “without” means no restraints were used. The parameters for restraints are the following: Distance restraints {$\kappa _{dr} = 1000$ kJ mol$^{-1}$ nm$^{-2}$;$\tau _{dr} = 500$ ps},dihedral restraints $\kappa _{dihr} = 1000$ kJ mol$^{-1}$ rad$^{-2}$, and orientation restraints $\kappa _{or} = 10$ kJ mol$^{-1}$ Hz$^{-2}$

* To be able to analyse the orientation restraints violation we had to include them into the simulations, but the corresponding force constant was 3 order of magnitude lower than the one we usually use

### *De novo *refinement and folding

**Fig. 1:**
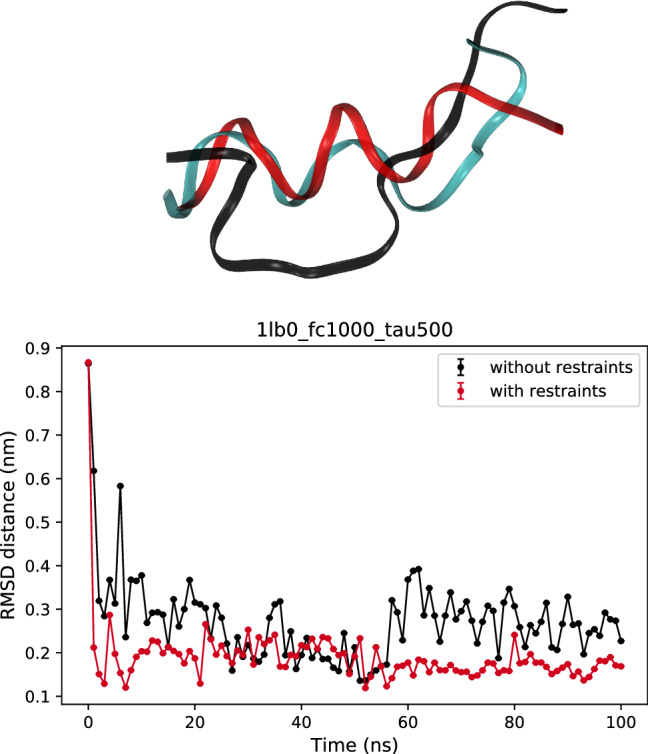
Folding of 1lb0. Top: comparison of the structures after 100 ns of simulations. The native conformation stated in the original PDB structure (Biron et al. 2002) (cyan), with (red) and without (black) restraints. We used VMD software for the visualisation (Humphrey et al. 1996). Bottom: The distance RMSD as a function of time for simulations with (red) and without (black) restraints. RMSD is calculated against the native conformation shown at the top

For one of the peptides a *do novo* refinement was attempted where the initial conformation for the simulation is completely extended. The folding of 1lb0 in simulations with and without restraints is shown in Figure 1 using the distance RMSD as a function of time. The reference frame for RMSD calculation is the original PDB structure. The largest change in structure occurs at the beginning both simulation, where the protein is fully denatured. It can be concluded that taking into account restraints provide a faster and more robust approach to obtaining the native conformation as well as more stable structure. Nevertheless, the final structure of the restrained simulation still differs somewhat from the reference structure. This could be due to the difference in temperature, the NMR structure was derived at 277K, whereas our simulations were done at room temperature. Indeed, circular dichroism measurements show that the peptide is somewhat less structured at room temperature (Biron et al. 2002). The average distance restraint violation is 0.001 nm for simulations with restraints and 0.010 nm for “without restraints” simulations.

## Conclusion

In this contribution, we present a Python package for importing data from nuclear magnetic resonance files NMR-STAR files into the GROMACS software. We have examined 8 different polypeptides with distance, dihedral and orientation restraints (Table 2). From a comparison of the values of corresponding restraint violation from GROMACS simulations with the restraints and without them (Table 4), we conclude that the package treat the restraints correctly.

For distance restraints we suggest the following parameters for force constant and the averaging time: $k_{dr}$ = 1000 kJ mol$^{-1}$nm$^{-2}$ and $\tau _{dr}$ = 500 ps, based on the evaluation presented in Table 3. Another step in this research should be to find the optimal parameters for dihedral and orientation restraints in the same way as we have done for distance restraints, however it should be kept in mind that these parameters may be system dependent.

Finally, we have used GROMACS to refine the 1lb0 peptide structure from an extended conformation. Simulations with and without restraints were run and it was found (Figure 1 bottom) that simulations with restraints converge to the experimental structure faster and end up with lower violations. The better converge can also be seen in the 3D representation of the structures at the top of the Figure 1. The red protein was simulated with the restraints and it fits the original 1lb0 (cyan protein) much better that the black one which was simulated without the restraints. However, one can see that after 100 ns of the simulations even for simulations with restraints, the folding is not perfect.

## Data Availability

The software described here, including test data, is available free of charge under the Apache License 2.0 from GitHub (Sinelnikova et al. 2020).
